# Transforming hematology care in LMICs: strategies for enhanced diagnosis and treatment

**DOI:** 10.3389/fpubh.2026.1907331

**Published:** 2026-08-13

**Authors:** Virak Sorn

**Affiliations:** Faculty of Health Sciences and Biotechnology, University of Puthisastra, Phnom Penh, Cambodia

**Keywords:** blood transfusion services, diagnostic capacity, hematology care, low- and middle-income countries (LMICs), universal health coverage (UHC)

## 1 Introduction

Hematologic disorders constitute a growing public health challenge in low- and middle-income countries (LMICs), where the burden of anemia, sickle cell disease (SCD), thalassemia, and many hematologic malignancies remains disproportionately high because of demographic, epidemiologic, and health system factors (1–3). Healthcare systems in many LMICs continue to face persistent challenges, including inadequate infrastructure, shortages of skilled healthcare professionals, limited diagnostic capacity, and constrained health financing, all of which impede timely diagnosis and effective management. Consequently, hematologic disorders, including anemia, SCD, thalassemia, leukemia, and lymphoma, remain major contributors to morbidity and mortality, particularly among vulnerable populations such as women, children, and underserved communities (2–4).

Despite major advances in hematology diagnostics and therapeutics globally, many LMICs continue to experience limited access to laboratory services, blood transfusion systems, specialist care, and essential medicines (2, 5). Delayed diagnosis and treatment interruptions frequently lead to preventable complications, disease progression, and poor survival outcomes. Furthermore, the increasing burden of non-communicable diseases, infectious diseases, malnutrition, and population aging has intensified pressure on already fragile healthcare systems across LMICs (6).

As many LMICs have only a handful of specialists, who are often overburdened and concentrated in major cities. This has created a cycle of inadequate training and mentorship for the next generation of health care providers, further compromising the ability of health systems to manage hematologic diseases (3, 7). Strengthening hematology care is therefore essential for improving health equity and advancing universal health coverage (UHC). Therefore, this viewpoint discusses the burden of hematologic diseases in LMICs, then highlights key diagnostic and treatment challenges, and proposes strategic interventions and regional approaches to improve hematology services.

## 2 Burden of hematologic diseases in LMICs

### 2.1 Nonmalignant hematologic disorders

Nonmalignant hematologic disorders impose a substantial public health burden across LMICs, particularly among women, children, and underserved populations. Anemia remains one of the leading causes of disability worldwide and disproportionately affects individuals in resource-limited settings because of iron deficiency, chronic infections, malaria, HIV, tuberculosis, and nutritional deficiencies (8, 9). Inherited red blood cell disorders also contribute substantially to morbidity and premature mortality. Approximately 75% of global SCD births occur in sub-Saharan Africa, where newborn screening and comprehensive care services remain limited (10). Likewise, thalassemia is highly prevalent in South Asia, Southeast Asia, the Middle East, and parts of the Mediterranean region, placing considerable demands on healthcare systems because of lifelong transfusion requirements and iron chelation therapy (10–12). Although the management of these disorders generally relies on less resource-intensive interventions than hematologic malignancies, effective care still depends on early diagnosis, reliable laboratory services, safe blood transfusion systems, uninterrupted access to essential medicines, and structured long-term follow-up. Without timely diagnosis and appropriate treatment, these conditions frequently result in severe complications, disability, and premature mortality.

### 2.2 Nonmalignant hematologic disorders

Hematologic malignancies, including leukemia, lymphoma, and multiple myeloma, are increasingly recognized in LMICs as diagnostic capacity improves and populations age (11). Despite their lower prevalence compared with nonmalignant hematologic disorders, these cancers are associated with substantial morbidity, mortality, and financial hardship because their diagnosis and management require sophisticated pathology services, flow cytometry, cytogenetics, molecular diagnostics, multidisciplinary specialist care, and access to increasingly expensive targeted therapies. Survival outcomes remain considerably lower than those observed in high-income countries owing to delayed diagnosis, inadequate pathology infrastructure, limited access to chemotherapy and novel therapeutics, shortages of trained specialists, and insufficient supportive care (4, 11–14). Consequently, patients and their families often experience catastrophic out-of-pocket healthcare expenditures and prolonged treatment-related financial hardship.

Antimicrobial resistance (AMR) further compounds these challenges, particularly among immunocompromised patients receiving intensive chemotherapy or hematopoietic stem cell transplantation. Resistant bacterial and fungal infections are increasingly recognized as major contributors to treatment-related morbidity and mortality in LMICs, where limited access to microbiological diagnostics, antimicrobial stewardship programs, and infection prevention measures further compromises clinical outcomes (15–17).

### 2.3 Challenges in diagnosis and treatment of hematologic disorders in LMICs

One of the major barriers to hematology care in LMICs is inadequate diagnostic infrastructure. Many healthcare facilities lack essential laboratory technologies, including automated hematology analyzers, hemoglobin electrophoresis, coagulation testing, flow cytometry, and molecular diagnostics (4, 5). Diagnostic services are frequently concentrated in urban tertiary hospitals, leaving rural and remote populations with limited access to specialized testing.

Blood transfusion services also remain fragile in many LMICs. Weak donor recruitment systems, inadequate blood screening, and limited cold-chain infrastructure contribute to blood shortages and increase the risk of transfusion-transmissible infections (18). Patients requiring regular transfusions, including those with SCD and hematologic malignancies, are particularly vulnerable to these challenges.

Shortages of hematologists, hematopathologists, oncology nurses, and laboratory scientists further undermine service delivery (1, 2). Existing specialists are often concentrated in major urban centers, resulting in substantial geographic disparities in care access (18, 19). In addition, essential medicines such as hydroxyurea, iron chelation therapy, chemotherapy, and clotting factor concentrates are frequently unavailable or unaffordable because of weak procurement systems and limited insurance coverage (18).

### 2.4 Strategic interventions for transforming hematology care in LMICs

Transforming hematology care in LMICs requires integrated and sustainable health system reforms. Strengthening laboratory infrastructure should be prioritized through the development of tiered laboratory networks linking primary, secondary, and tertiary healthcare facilities (5). Expanding point-of-care diagnostics and regional referral laboratories may improve access to timely and accurate diagnosis.

Improving equitable access to essential medicines and innovative hematology therapies remains a critical priority for LMICs. National essential medicines lists should include key hematology treatments aligned with World Health Organization recommendations while ensuring their sustainable availability through strengthened procurement and supply chain systems. Regional pooled procurement mechanisms and public-private partnerships can improve medicine affordability, reduce supply chain disruptions, and increase negotiating power with pharmaceutical manufacturers (18–21). In addition, industry-supported access initiatives, including differential pricing strategies, compassionate-use programs, treatment donation initiatives, patient assistance programs, and outcome- or revenue-based access agreements, may facilitate access to high-cost therapies, particularly for patients with hematologic malignancies who require novel targeted agents or cellular therapies (3, 13, 14, 20). To ensure long-term sustainability and equitable access, these mechanisms should be transparent, integrated within national health financing frameworks, and implemented through partnerships among governments, healthcare providers, pharmaceutical manufacturers, and international organizations rather than relying solely on short-term charitable initiatives (13, 14, 20). Strengthening blood transfusion services through voluntary blood donation campaigns, improved screening technologies, robust quality assurance systems, and reliable cold-chain infrastructure remains equally essential for supporting comprehensive hematology care (18).

Strong government leadership is fundamental to ensuring the long-term sustainability of hematology services in LMICs. National governments should prioritize hematology within health sector strategies by increasing domestic investment, integrating essential hematology services into UHC benefit packages, and establishing sustainable procurement mechanisms for diagnostics, blood products, and essential medicines (1, 19–21). Strategic partnerships with non-governmental organizations (NGOs), academic institutions, international agencies, and development partners can further strengthen workforce capacity, laboratory infrastructure, technology transfer, medicine procurement, and implementation of sustainable hematology programs (21). Such multisectoral collaborations should complement national leadership and be aligned with country priorities to promote long-term health system resilience and equitable access to quality hematology care.

For patients requiring highly specialized diagnostic procedures or treatments that are unavailable locally, formally established regional and international referral networks can play a critical role in ensuring equitable access to advanced hematology care (22). Such referral pathways are particularly important for patients with complex hematologic malignancies who require hematopoietic stem cell transplantation, cellular therapies, advanced molecular diagnostics, or other highly specialized interventions (23, 24). Whenever feasible, these referral systems should be supported by formal institutional agreements that clearly define patient eligibility criteria, financing mechanisms, shared clinical responsibilities, continuity of care, data sharing, and post-treatment follow-up (25). Establishing structured referral networks, rather than relying on *ad hoc* referrals, can improve care coordination, reduce inequities in access, optimize resource utilization, and strengthen regional collaboration in hematology services across LMICs.

Workforce development initiatives, including postgraduate hematology training, continuing professional education for clinicians, laboratory personnel, and allied health professionals, may help address specialist shortages (26). Digital health technologies also present important opportunities for LMICs to strengthen healthcare access (27). Telehematology platforms can support remote consultations, specialist mentoring, blood smear interpretation, and follow-up care, particularly in geographically isolated areas (6, 27–29). Electronic health records and disease registries may further strengthen surveillance systems and continuity of care.

Research and data generation are also essential for advancing hematology care in LMICs. Many countries lack reliable epidemiological data on hematologic diseases, leading to under-prioritization within national health agendas (1–4). Limited research funding, weak infrastructure, and insufficient institutional capacity further constrain local evidence generation. Strengthening hematology research through regional collaborations, international partnerships, and dedicated funding mechanisms is critical for informing context-specific policies and evaluating intervention effectiveness.

## 3 Regional experiences in hematology care across LMICs

Regional experiences across LMICs demonstrate both the substantial burden of hematologic disorders and the diverse strategies being implemented to strengthen hematology care. Sub-Saharan Africa continues to carry one of the highest global burdens of SCD and severe anemia, accounting for approximately 75% of global SCD cases (10–12). Several countries, including Nigeria, Ghana, Kenya, Uganda, and Tanzania, have introduced newborn screening programs, hydroxyurea treatment initiatives, and laboratory strengthening strategies to improve hematology services (11, 20).

In South Asia, anemia, thalassemia, and hematologic malignancies remain major public health concerns driven by nutritional deficiencies, inherited blood disorders, and socioeconomic disparities (30). Efforts to integrate hematology services into national non-communicable disease strategies and essential health service packages may improve healthcare access, financial protection, and continuity of care (21). Meanwhile, countries in the Middle East and North Africa region have strengthened premarital screening programs, genetic counseling, and public awareness campaigns to improve prevention and early diagnosis of inherited blood disorders such as thalassemia and SCD (31).

Across East Asia and the Pacific, telemedicine and digital health technologies are increasingly being utilized to address workforce shortages and improve access to hematology specialists (18). Telehematology platforms support remote consultations, blood smear interpretation, specialist mentoring, and continuing medical education, particularly in geographically isolated areas. Similarly, several LMICs in Europe and Central Asia have focused on strengthening rehabilitation and supportive care services for patients living with chronic hematologic disorders through community-based rehabilitation, psychosocial support, and integrated chronic disease management approaches (30).

In Latin America and the Caribbean, disparities in access to specialized hematology services, variability in diagnostic capacity, and limited epidemiological data continue to hinder evidence-based healthcare planning and equitable delivery of hematology care (32). Although several countries have made progress in strengthening surveillance systems, laboratory networks, and regional research collaborations, substantial gaps persist in the availability, accessibility, and quality of hematology services. These limitations contribute to delayed diagnosis, fragmented care, and suboptimal management of hematologic disorders. Furthermore, the growing burden of cancer has been identified as one of the leading causes of mortality across the region, highlighting the urgent need to strengthen hematology and oncology services through integrated health system approaches (32–34). Continued investment in national surveillance systems, laboratory infrastructure, workforce capacity, and regional research collaborations will be essential to improving hematology care delivery, generating locally relevant evidence, and informing context-specific policies across LMICs.

## 4 Conclusion

Hematologic disorders continue to impose a substantial burden across LMICs, where healthcare systems face persistent challenges in diagnostics, treatment access, workforce shortages, and blood safety. Strategic investments in laboratory strengthening, blood transfusion systems, workforce development, digital health technologies, and essential medicines are essential for improving hematology care outcomes. Integrating hematology services into broader UHC and health system strengthening initiatives may improve sustainability and health equity. Long-term progress will require strong political commitment, equitable financing, regional collaboration, and international partnerships to ensure accessible and high-quality hematology care for vulnerable populations in LMICs.
